# Glycosylation of viral proteins: Implication in virus–host interaction and virulence

**DOI:** 10.1080/21505594.2022.2060464

**Published:** 2022-04-18

**Authors:** Tingting Feng, Jinyu Zhang, Zhiqian Chen, Wen Pan, Zhengrong Chen, Yongdong Yan, Jianfeng Dai

**Affiliations:** a Jiangsu Key Laboratory of Infection and Immunity, Institute of Biology and Medical Sciences, Soochow University, Suzhou, China; b Department of Respiratory Medicine, Children’s Hospital of Soochow University, Soochow University, Suzhou, China

**Keywords:** Glycosylation, viral structural protein, viral nonstructural protein, virus life cycle, viral pathogenesis

## Abstract

Glycans are among the most important cell molecular components. However, given their structural diversity, their functions have not been fully explored. Glycosylation is a vital post-translational modification for various proteins. Many bacteria and viruses rely on *N*-linked and O-linked glycosylation to perform critical biological functions. The diverse functions of glycosylation on viral proteins during viral infections, including Dengue, Zika, influenza, and human immunodeficiency viruses as well as coronaviruses have been reported. *N*-linked glycosylation is the most common form of protein modification, and it modulates folding, transportation and receptor binding. Compared to *N*-linked glycosylation, the functions of O-linked viral protein glycosylation have not been comprehensively evaluated. In this review, we summarize findings on viral protein glycosylation, with particular attention to studies on N-linked glycosylation in viral life cycles. This review informs the development of virus-specific vaccines or inhibitors.

## Introduction

The high number of infectious diseases caused by viruses has posed a major threat to global public health [1]. Moreover, there are no vaccines and antiviral drugs for most of the newly emerged viruses. Therefore, there is an urgent need to develop antiviral strategies to control the spread of these viral pathogens.

Viruses are obligate intracellular infectious agents that exploit host machinery to modify their viral proteins for survival. One of the key modifications is protein glycosylation. Glycosylation is the attachment of glycans to proteins and is a critical post-translational modification (PTM). To date, various glycosidic linkages such as *N*-, O-, and C-linked glycosylation, glypiation (GPI anchor attachment), and phosphoglycosylation have been reported [2]. Among them, *N*-linked glycosylation, where glycans are attached to the amide nitrogen of asparagine (Asn), is the most common and extensively studied form of protein glycosylation [3].

Recent studies have shown that many viral proteins, especially structural proteins are glycosylated during viral infection cycles. The *N*-glycans of the viral glycoproteins have multiple functions, which include promotion of expression, transport, fusion, binding to cell surface receptors, and prevention of antibody neutralization. For instance, glycosylation of envelope proteins of the human immunodeficiency virus (HIV), Japanese encephalitis virus (JEV) and West Nile virus (WNV) is essential for membrane fusion and virus infectivity [4–6]. Similarly, Influenza virus (IAV), lymphocytic choriomentingitis virus (LCMV), Lassa virus (LFV), Severe acute respiratory syndrome virus (SARS), Zika virus (ZIKV), Dengue virus (DENV) and Ebola virus (EBOV) also have their extensively glycosylated envelope proteins [7–13] (Table 1). Furthermore, some host cell-derived glycans have diverse effects in the life cycle of viruses, including glycan shield mediated immune evasion and enhancement of immune cell infection [14]. Here, we analyze recent findings on the importance of glycosylation to viral infection and host immune response.

**Table 1.** Current understanding of glycosylation of viral proteins

Virus	Viral Protein	Glycosylation site	Potential function	References
DENV	Envelope	Asn-67	Transmission, Replication, Virus entry	[11,22]
		Asn-153	Survival in mosquito	[11,21,22]
	NS1	Asn-130	Virus growth, NS1 secretion, Cytopathy and neurovirulence	[21,144–147]
		Asn-207	Destroy endothelial layer and internalization	[21,142,143]
ZIKV	Envelope	Asn-154	Replication, Assembly, Infectivity, Neurovirulence, Apoptosis, Invasion in mosquito	[28–31]
	prM	Asn-69	Virus production, Expression and secretion of ZIKV E	[12]
	NS1	Asn-130 and Asn-207	Internalization and endothelium barrier function	[12]
WNV	Envelope	Asn-154	Assembly and infectivity of particle, Replication and infection in mosquito, Neuroinvasiveness in mice	[6,36–39]
	prM	Asn-15	Assembly and release of particle	[6]
	NS1	Asn-130, Asn-175 and Asn-207	Internalization and neuroinvasiveness in mice	[150]
JEV	Envelope	Asn-154	Replication, Neurovirulence and neuroinvasiveness in mice	[40,41]
	prM	Asn-15	prM protein Biogenesis, Virus particle release, Pathogenicity in mice	[5]
HCV	E1 and E2		Protein folding, Virus entry, Immune response, Assembly and/or secretion of viral particle	[42–45]
HIV	Env/gp120	Asn-260	Expression of gp120 and gp41, Infectivity and virus entry	[4,52–54]
IAV	HA and NA		Protein folding, transport and pH stability; Affinity of SA receptor and viral pathogenicity; Functional NA and neurovirulence in mice; Receptor binding, Infectivity, Virus release, and neurotoxicity	[56–67]
SARS-CoV-2	Spike	Asn-90 or Asn-322	Virus entry	[10,71]
EBOV	GP1		Transduction of viral particle, Sensitivity to Cathepsin B	[13,79]
	GP2	Asn-563 and Asn-618	Virus entry	[79]
SFTSV	Gn	Asn-33 and Asn-63	Immune response	[80,81]
	Gc	Asn-853, Asn-914 and Asn-936	Immune response and membrane fusion	[80,82]
HTNV	Gn	Asn-134, Asn-235, Asn-347, Asn-399 and Asn-609	Folding, Intracellular transport and epitope conformation; Maintenance of HTNV glycoprotein; Immunoreactivity	[85,86]
	Gc	Asn-928	Immunoreactivity	[86]
RVF	Gn	Asn-438	Infection and inducing neutralizing antibodies	[87–89]
	Gc	Asn-794, Asn-829, Asn-1035, and Asn-1077	Infection and inducing neutralizing antibodies	[87–89]
NiV	F and G		Virus entry, Antibody neutralization and cell-cell fusion	[92,93]
HeV	F and G		Virus attachment and membrane fusion	[94]
HRSV	F	Asn-27, Asn-70, Asn-116, Asn-126 and Asn-500	Attachment, Replication and Infection; Syncytium formation and antigenicity of F protein	[95–97]
	G		Membrane fusion	[95]
LCMV	GP1	Asn-87, Asn-97, and Asn-104	Folding of GPC and virus adaptability	[8,99,100]
	GP2	Asn-234 or Asn-379/381	Membrane fusion and virus adaptability	[8,100]
LASV	GP1/GP2	Asn-89, Asn-99, Asn-109, Asn-119, Asn-167, Asn-365 and Asn-373	Envelope glycoprotein cleavage, Infectivity and immune response	[9,102–107]
HBV	S	Asn-146	Production of infectious virus particles	[108]
HSV-1	gB, gC, gD, gE gH/gL, gK, gI		Attachment, Penetration, Membrane fusion, Receptor interactions; Immunity interference, Pathogenesis, Production of noninfectious virus particles, Infectivity	[111,113–119,126–128]
VZV	gB, gC, gE, gH, gI, gK, and gL		Assembly,Trafficking, Reproduction, Replication, virion-cell, cell-cell fusion, Cell-to-cell spread and infectivity	[129–133]

## The roles of glycosylation on viral structural proteins

### Glycosylation and its function on flavivirus structural protein

**Figure 1.**
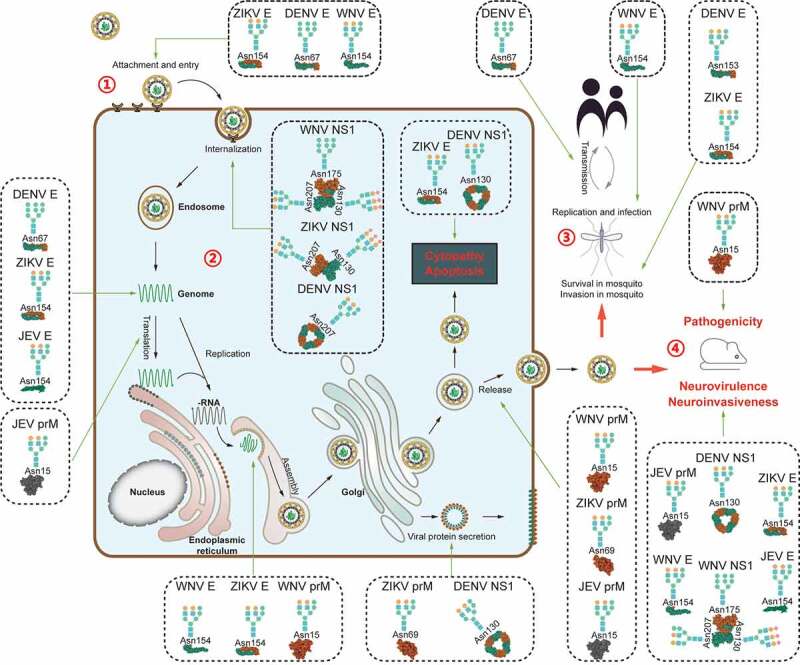
Diverse functions of glycosylation of viral proteins from mosquito-borne flaviviruses.

Note: The structural proteins (E and prM) and nonstructural protein NS1 of flaviviruses are glycosylated and play multiple roles in the entire lifecycle of viruses. 1) Glycosylations of viral proteins influence virus binding and evasion. 2) Glycosylation of viral protein affects viral lifecycle in cells, including replication, translation, assembly, and release. 3) Glycosylations of viral proteins play a role in viral survival and transmission in mosquitoes. 4) Glycosylation of viral proteins determines viruses’ pathogenicity in mammalian hosts. The 3-D models used for representing the flavivirus protein structures were cited from the PDB database (PDB accession numbers: ITG8, 4OIG, 2HG0, 4O6C, 5MV1, 5O36, 5JHM, 3C5X, and 5K6K). The glycan chains represented here are hypothetical schematic sketches.

Dengue virus (DENV), a member of the flavivirus genus of the Flaviviridae family, causes the most common arthropod-borne viral diseases in humans, and poses a huge threat to the public social, health, and economic status [15]. DENV has four different serotypes (DENV-1, DENV-2, DENV-3, and DENV-4), all of which can cause disease [16,17]. The viral particles are composed of three structural proteins, namely capsid (C), envelope (E), and (pre) membrane protein (prM/M) [18–20]. In addition, DENV has seven nonstructural (NS) proteins, which include NS1, NS2A, NS2B, NS3, NS4A, NS4B, and NS5 [21]. Numerous studies have shown that the E protein plays an essential role in host receptor attachment, cellular uptake of viral particles, and membrane fusion [17,21]. The E protein has two potential *N*-linked glycosylation sites at Asn-67 and Asn-153. The Asn-153 glycosylation site is conserved in most flaviviruses, while the Asn-67 glycosylation site is unique to DENV[11](Figure 1). The glycosylation of envelope E protein at Asn-67 has an important role in the transmission of DENV [11]. Absence of Asn-67 glycosylation significantly suppresses replication of DENV and reners the virus unable to produce new infectious particles [11]. Besides, Asn-67 glycosylation of the DENV E protein was also shown to significantly enhance the entry of DC-SIGN-mediated dengue virus [22,23]. On the other hand, *N*-glycans located at Asn-153 of the DENV E protein are beneficial in the survival of DENV in both mammalian and mosquito cells [11,21,22]. In DENV lifecycle, the prM/M protein was also shown to be *N*- glycosylated at amino acid positions 7, 31, 52, and 69 [21,24]. However, the functions of prM/M glycosylation have not been extensively studied [21].

Zika virus (ZIKV) is an emerged mosquitoe-borne flavivirus that causes severe human diseases, which include neurodevelopmental malformations (congenital Zika syndrome) and Guillain-Barre syndrome [25–27]. The prM and E proteins of the ZIKV virus have glycosylation sites. The *N*-linked glycosylation at Asn-154 of the ZIKV E protein was shown to affect virus assembly and infectivity *in vitro* [28], and also mediate invasion of the ZIKV into the mosquito midgut [29] (Figure 1). The *N*- linked glycosylation of the ZIKV E protein may enhance viral infectivity through cell surface lectins [28,30]. Fontes-Garfias et al demonstrated that in type 1 interferon-deficient mice and mosquito cells, *N*-linked glycosylation at Asn-154 was essential for ZIKV infection but does not affect the neurovirulence in mice [30]. However, in Asian ZIKV strain ZKC2P6, the N154Q mutation or deletion at the N154 position of E protein facilitated the ZIKV replication and enhances neurotoxicity and apoptosis in newborn mice [31] (Figure 1).

N-linked glycosylation of the prM protein is also essential for the life cycle of the ZIKV. The prM protein of all the ZIKV strains contains a single N-linked glycosylation site at Asn-69^12^. A previous study showed that loss of prM N-glycans leads to protein aggregation and induce CHOP nuclear translocation [12]. Furthermore, prM and E N-glycans in the ZIKV are essential for effective secretion of the ZIKV E protein, and both are indispensable [12]. Lack of prM N-glycosylation leads to impaired expression and secretion of the E protein, which causes accumulation of the E protein in the endoplasmic reticulum (ER), thus triggering ER stress response [12], a phenomenon that is detrimental to the ZIKV life cycle.

West Nile virus (WNV) is also an arthropod-transmitted virus that belongs to the Flaviviridae family. Although the prM proteins of all the WNV strains contain *N*-linked glycosylation sites, but not all strains contain an *N*-linked site in the E protein [6]. The *N*-linked glycosylation motif (NYS/T) of the WNV E protein is located between amino acid positions 154–156 [32–35]. Martina et al showed that the WNV strains containing E glycosylation at the N154 can use DC-SIGN as an attachment factor to enhance virus infection [36]. In other studies, WNV lacking N-linked glycans in the E protein could not efficiently replicate and spread in mosquito cells [37,38]. Once the WNV E protein is glycosylated, virus assembly is enhanced and the viral infectivity is significantly increased [6]. On the other hand, when mice were infected with the WNV, only the virus with glycosylated E protein showed neuroaggressiveness, suggesting that glycosylation of the E protein is a molecular determinant of the WNV neuroaggressiveness [39]. The prM protein of WNV has a potential N-linked glycosylation site at amino acid 15 of the extracellular domain [6]. Studies have shown that N-linked glycosylation sites in the WNV prM play a role in regulating virus assembly and release, but have little effect on virus infectivity. Removal of glycosylation on the prM or E protein results in reduction of the release of subviral particles [6]. Nevertheless, the specific role(s) of glycosylation in WNV pathogenesis needs further evaluation.

The two membrane glycoproteins of Japanese encephalitis virus (JEV), prM and E, contain an *N*-linked glycosylation site at positions N15 and N154, respectively [5,40]. In animal model studies, removal of prM *N*-glycosylation resulted in a sharp decline in viral virulence in mice after virus inoculation [5]. With mutation of *N*-linked glycosylation site of prM, the growth of JEV is inhibited in a cell-type-specific manner [5]. In a DNA vaccine study of JEV, immunization of proteins with both prM and E glycosylation site mutations were shown to significantly enhance the antibody response, increase IL-4 secretion, and provide complete protection against lethal attack of the JEV [40]. In addition, Liang et al. employed three types of JEV mutants to determine the role of glycosylation of JEV E protein in viral pathogenesis [41]. Compared with the N154 glycosylated wild-type JEV, strains with no glycosylation or N67 glycosylation showed less growth fitness, and decreased neurovirulence/neuroinvasion in mice. Furthermore, compared with the wild-type viruses, viruses with both N67 and N154 E glycosylation showed effective replication and neurovirulence in cell culture, but reduced neuroinvasiveness *in vivo* (Figure 1). Therefore, the JEV E protein with a single glycosylation site at N154 was likely to have the selection advantage in crossing the blood–brain barrier during evolution [41]. This study also supports the role of flavivirus E protein glycans on viral infections and cell tropism.

Hepatitis C virus (HCV) encodes two envelope glycoproteins E1 and E2. There are 4 and 11 *N*-linked glycosylation sites on E1 and E2, respectively [42,43]. They play an important role in protein folding, virus entry, and regulation of immune responses [42–45]. Mutations in glycosylation sites E1N1, E2N3, E2N7, E2N8, E2N10, and E2N11 have a negative impact on virus particle assembly and/or secretion as well as virus entry [42]. Moreover, the E2N7 mutation greatly reduces the infectivity of HCV. In addition, the glycans at the E2N2 and E2N4 positions are essential for the entry function of the HCV envelope glycoproteins. Glycans at positions E2N1, E2N2, E2N4, E2N6, and E2N11 reduced the accessibility of neutralizing antibodies to their E2 glycoprotein epitopes. Besides, the glycans at E2N1, E2N2, E2N4, and E2N6 could regulate binding between CD81 and E2, which may partly explain how HCV evades the immune response [42].

For most flaviviruses, glycosylation of the envelope protein affects the adaptability, infectivity, replication, and virulence of the virus [6,11,46–51]. To date, the structure and functions of these glycans in virus transmission, infection and pathogenesis are not fully understood yet, and further exploration is needed.

### Glycosylation of HIV envelope protein

The human immunodeficiency virus (HIV) envelope glycoprotein (Env) is composed of surface subunit gp120 and transmembrane subunit gp41 [4]. It is reported that the *N*-linked glycans on the surface of gp120 are essential for proper protein folding. HIV gp120 contains nine disulfide bonds and is highly glycosylated, carrying an average of 24 *N*-linked glycans. Among them, the Asn260 glycosylation site defines correct expression of gp120 and gp41 [4]. Studies have shown that N260Q mutation could affect the folding and lysosomal degradation of the gp120, leading to loss of viral infectivity. Further studies have shown that the reduction of virus infectivity is caused by the deletion of glycans in the gp120 V1/V2 domain [4,52]. Moreover, several highly conserved *N*-linked glycans of the gp120 are preferentially located near the disulfide bridge. Removal of disulfide bonds has been shown to significantly affect the ability of the DC-SIGN to bind to HIV. On the other hand, in a variety of virus strains lacking N-glycans, introduction of new N-glycans upstream of several disulfide bonds could result in complete loss of viral infectivity [53]. The ability to bind to the CD4 receptor was significantly reduced in the gp120 strain bearing the N260Q mutation, suggesting that N260 glycosylation affects the virus entry process [54]. In addition, N-linked glycans could affect protein function and neutralizing antibody response [55]. Given the pivotal role of N-glycans in HIV-1 gp120 V1/V2 domains, most studies have focused on neutralizing antibody response for gp120 N-linked glycans, which could be a new target for specific therapeutic drug intervention.

### Glycosylation and biological functions of IAV structural protein

Influenza A virus (IAV) encodes two main viral surface glycoproteins, hemagglutinin (HA), and neuraminidase (NA). The HA protein determines the antigenicity of the IAV. The HA of most human H1N1 has multiple *N*-glycosylation sites ranging from 5 to 11, most of which are located in the globular head of the HA molecule [14,56]. Glycosylation of the HA stem region is important for protein folding, transport, and pH stability [57–59]. Other studies have shown that change of glycosylation near HA receptor binding site would change its affinity to receptors [60–62]. Glycosylation near the cleavage HA cleavage site also regulates the pathogenicity of the virus [63,64]. Furthermore, the loss of a single *N*-linked glycan in HA was associated with the related to the resistance to collection and increased virulence in mice [65]. *N*-linked glycosylation is also important in the NA functions [66]. Lack of NA glycosylation could increase the neurovirulence of mouse A/WSN/33 IAV strain [67].

### Glycosylation and biological functions of Coronaviruses structural proteins

SARS-CoV-2 is a novel virus that has caused the global pandemic “Coronavirus Disease2019” (COVID-19), a severe acute respiratory disease [68]. The viral Spike protein, its receptor binding domain (RBD) and its receptor ACE2 are all extensively glycosylated. The Spike protein contains 22 *N*-glycosylation sites, while ACE2 contains 7 *N*-glycosylation sites [69–71]. The O-linked and *N*-linked glycans of the SARS-CoV-2 Spike protein are less important in regulating the direct binding of the Spike and ACE2, but inhibit the virus entry [10]. Studies have shown that blocking of N-glycan biosynthesis with small-molecule inhibitors inhibits entry of the SARS-CoV-2. Besides, blocking the processing of O-glycans was shown to partially block the virus entry. Analysis of the crystal structure showed that the key glycans regulating this process could be located at Asn-90 or Asn-322 position of the Spike protein [72]. However, the detailed mechanisms for this site-specific glycosylation regulating the entry of SARS-CoV-2 remain unclear.

Some coronaviruses have E1 and E2 envelope proteins. Previous data showed that E1 protein is an O-linked glycoprotein while E2 is *N*-linked glycosylated [73–76]. The M protein of coronavirus is the most abundant protein, and plays a central role in virus assembly. SARS-CoV M protein contains an *N*-glycosylation site at N4 [76,77]. Although the glycosylation of the coronavirus M protein is a highly conserved feature, this glycosylation is not important for viral assembly or replication [78].

### Glycosylation and biological function of EBOV structural protein

Ebola virus (EBOV) belongs to the filovirus family, and its surface glycoprotein (GP) is composed of trimers of GP1/GP2 heterodimers. The GP1 subunit is important for receptor binding, while the transmembrane-related GP2 subunit is necessary for membrane fusion. EBOV GP1 contains 15 *N*-glycosylation sites. A previous study showed that loss of any of *N*-glycosylation sites does not affect the expression of GP but enhance pseudovirion transduction [13]. Removal of GP1 *N*-glycans does not affect the binding of pseudoviral particles to the cell surface, but increases protease sensitivity of the cathepsin B [13]. In antibody neutralization assays, elimination of N-linked glycans in GP1 can significantly increase the sensitivity of antibody neutralization, while introducing N618D to the GP1 subunit (7Gm8 G), which could further enhance neutralization sensitivity of the virus particles [13,79]. The GP2 subunit of filovirus contains two highly conserved N-linked glycosylation sites at N563 and N618 positions [79]. Deletion of the glycosylation site at N563 could lead to enhanced virus entry, possibly through partial destruction of the stability of the GP. Removal of a single glycan at N563 or N618 could not increase the sensitivity to neutralizing antibodies. Besides, antibody sensitivity is increased in viruses lacking all the N-linked glycans in the GP1 or the glycan at N618 in the GP2, compared with viruses with GP1 single mutation [79].

### Glycosylation and biological functions of bunyavirus structural protein

Severe fever with thrombocytopenia syndrome virus (SFTSV) is a tick-transmitted bunyavirus with a segmented, negative-strand RNA genome, which includes large (L), medium (M), and small (S) segments. The M segment encodes a membrane protein precursor that matures into two glycoproteins, Gn and Gc. Recent studies have shown that SFTSV-specific neutralizing antibodies, mainly induced by Gn and Gc, play a vital role in the protective immune response [80]. Gn has two *N*-linked glycosylation sites, at Asn 33 and Asn 63 [81]. SFTSV Gc is a type I transmembrane protein, whose extracellular domain is stabilized by 13 disulfide bonds and can form putative trimmers at acidic pH [82]. Analysis of the electron density of Gc has shown that there are three *N*-linked glycosylation sites in the extracellular domain of SFTSV Gc: Asn 853, Asn 914, and Asn 936^82^. DC-SIGN and Non-muscular myosin heavy chain IIA (NMMHC-IIA) have been reported to be the initial binding receptor for SFTSV [83,84]. Whether the *N*-linked glycans in Gn and Gc are critical for virus binding and entry remains to be defined.

Hantaan virus (HTNV) also encodes two glycoproteins, Gn and Gc. Gn contains five potential *N*-linked glycosylation sites at N134, N235, N347, N399, and N609 while Gc contains a potential *N*-linked glycosylation site at N928 [85]. All glycosylation sites on the extracellular domain of Gn (N134, N235, N347, and N399) and Gc (N928) are glycosylated, and the fifth Gn site (N609) which faces the cytosol remains unutilized [86]. N134 is most critical for guiding the correct folding of Gn. In the absence of this site, Gn is stagnated in the ER and cannot be recognized by anti-Gn MAb [86]. In addition, glycosylation at the third Gn site (N347) significantly affects its folding, while the lack of N235 and N399 sites in Gn and N928 site in Gc is insufficient to affect folding and targeting [86]. Besides, mutations of the N134, N347, and N399 *N*-glycosylation sites of Gn reduce the immunoreactivity of Gc protein. Conversely, lack of N-glycans on Gc has been shown to reduce immunoreactivity of the Gn protein [86].

Rift Valley Fever is a disease caused by Rift Vally Fever virus (RVFV) and is transmitted by mosquitoes. The RNA genome of the RVFV consists of large (L), medium (M), and small (S) segments. The M segment encodes the Gn and Gc glycoproteins. RVFV has five putative *N*-glycosylation sites, located at N438 of the Gn protein, and N794, N829, N1035, and N1077 of Gc protein, respectively [87]. Glycosylation at N438 of RVFV Gn or N1077 of the Gc plays an important role in mediating virus infection through DC-SIGN [87]. A previous study showed that in Jurkat-DC-SIGN cells, there was suppression of the infectivity of N438Q or N1035Q mutants, while N438Q/N1035Q double mutation had little effect on the viral infection of these cells [87]. Besides, RVFV Gn and Gc glycoproteins are crucial in inducing neutralizing antibodies [88,89].

### Glycosylation and biological functions of paramyxovirus structural protein

Nipah virus (NiV) is an emerging paramyxovirus with deadly consequences. NiV infects the respiratory and nervous systems and leads to fatal encephalitis, the main cause of human death [90,91]. The fusion (F) and attachment (G) envelope glycoproteins of the NiV mediate virus entry, cell–cell fusion, and syncytium formation. The NiV-F protein has five potential *N*-linked glycosylation sites, four of which are highly glycosylated [92]. Removal of *N*-glycans on F protein has little effect on protein processing and expression, but F3 and F5 *N*-glycan mutants have a higher fusion level compared to the wild-type NiV [92]. Removal of N-glycans in the NiV F protein results in significantly enhanced virus entry. Meanwhile, the N-glycans on NiV-F could protect the virus from neutralization by antibodies [92]. There are seven potential N-linked glycosylation sites in NiV-G, of which six are highly glycosylated [93]. The function of NiV-G N-glycan in membrane fusion is highly dependent on its environment, and some NiV-G N-glycans even reduce the fusion efficiency [93]. Like NiV, another paramyxovirus, Hendra virus (HeV), also requires two different membrane-anchored glycoproteins G and F for membrane fusion and viral entry [94]. The HeV-G head domain may have five N-linked glycosylation sites, which are partially occupied by carbohydrates [94]. To date, data on the N-linked glycosylation of these two HeV glycoproteins remain scant.

Human Respiratory Syncytial Virus (HRSV) belongs to the Pneumovirus genus, Paramyxoviridae. The virus envelope has two main glycoproteins, G and F, which are responsible for virus attachment to cells and membrane fusion [95]. The glycosylation sites in the G protein are highly variable, while those in the F protein are relatively conserved. The RSV F has five *N*-glycosylation sites, which are N27 and N70 located in the F2 subunit, N116 and N126 in the p27 peptide, as well as N500 in the F1 subunit. Every single *N*-glycosylation site in the RSV F protein is not dispensible for virus replication, but they have synergistic contribution to the efficiency of viral infection [96]. The virus can not replicate if all the *N*-glycans in the RSV F protein are removed. Data have demonstrated that the N-glycan at the N500 position is essential for syncytium formation in RSV-infected Hep-2 cells [96]. In addition, the frequency of syncytia formation in RSV F N116Q-infected cells was also significantly reduced, indicating that N-glycosylation at N116 affects syncytia formation. Another study showed that removal of the N-glycosylation site at position N116 can enhance the antigenicity of F protein [97]. The G protein contains potential N-glycosylation and O-glycosylation sites. Variations in the O-glycosylation are the main reasons for the high variation between virus strains and may contribute to immune evasion [96].

### Glycosylation and biological functions of arenavirus protein

Lymphocytic choroid meningitis virus (LCMV) belongs to the arenavirus family. There are three main structural proteins: one nucleocapsid protein (NP) and two glycoproteins (GP1 and GP2) [98]. AS previous study predicted nine potential *N*-linked glycosylation sites on the LCMV glycoprotein: 6 on GP1 and 3 on GP2 [8]. The three *N*-glycosylation sites on GP2 are conserved in all the members of this virus. In contrast, the number and location of *N*-glycosylation sites in GP1 are highly diverse. In addition, folding of the glycoprotein complex (GPC) precursor requires N-linked glycosylation in GP1 [99]. N-linked glycosylation site mutations of GP1 at positions 87, 97 and 104 impair the expression and processing of GPC [8]. Removal of glycans at position 234 or addition of glycans at position 379/381 in GP2 have been shown to impair GP-mediated cell fusion without altering expression or processing [8]. N-glycosylation mutations may affect VLP infectivity during viral fusion events. Mutations in N-glycosylation, at some sites reduce, the VLP infectivity, while the mutant S373A increases the infectivity. Among them, the N-glycan attached to the Asp173 mediates masking the neutralizing GP-1D epitope on Arm-5 antibody clone to prevent neutralization [8]. All three N-glycosylation mutants (T234A, S398A, and T403A) showed reduced adaptability in mouse macrophages. The rLCMV produced by N-glycosylation deletion mutant in GP1 (G104N) or GP2 (E379N/A381T) significantly reduced viral fitness in neurons [100]. These data indicated that LCMV glycosylation regulates viral fitness and cell tropism, and affects the viral growth.

Lassa virus (LASV), a member of the arenavirus family, causes Lassa fever [101]. LASV GP1 is responsible for attachment of the virus to the host cell receptor, while GP2 mediates the fusion of the virus and the endosomal membrane [102,103]. There are 11 *N*-glycosylation motifs in GPC, including 7 *N*-glycans on GP1 and 4 *N*-glycans on GP2 [9]. The glycosylation of GP1/GP2 is necessary for their transport, antigenicity, and infectivity [8,104,105]. Recent studies have shown that 25-hydroxycholesterol (25HC) could decrease the maturation of N-glycan of LASV glycoproteins, thus reducing production of infectious virus [106]. N-glycosylation of LASV glycoprotein requires STT3B, which is essential for viral infectivity [107]. In pseudotyped LASV, the N-linked glycosylation site mutants at N89 and N365 were shown to completely inhibit infectivity, while removal of N-linked glycans at positions N109 and N119 only partially inhibited the infectivity [9]. Besides, the specific glycan chains affect functions of CD4+ T cells and CD8+ T cells in splenic lymphocytes and help LASV escape the immune response by reducing the host’s recognition of GPC and preventing induction of immune response [9]. The role of N-glycosylation at 3^rd^ (N99), 5^th^ (N119), 6^th^ (N167), 8^th^ (N365) and 9^th^ (N373) sites can mask key epitopes in the GPC and escape humoral immune response [9]. These GPC sites may be targets for the development of effective therapeutic or preventive antibodies against Lassa fever.

### Glycosylation and biological functions of HBV envelope protein

Hepatitis B virus (HBV) is a human DNA virus that encodes three envelope glycoproteins, small (S), medium (M), and large (L). These three proteins share a potential *N*-glycosylation site at Asn-146 (N146) of their S domain [108,109]. Early *N*-glycan modification on the ER has a major impact on the production of infectious virus particles [108,110]. The hyper-glycosylated antigens induce earlier and longer-lasting humoral immune responses compared to the wild-type. *N*-glycosylation may also be a predictive marker for increased risk for cancer in patients with chronic HBV infection, which deserves more evaluation [108]. Although many features of the HBV life cycle are associated with viral glycosylation, the role of N-glycosylation in the pathogenesis of the virus remains unknown.

### Glycosylation and biological functions of Herpes virus and other human virus proteins

Herpes virus is a large enveloped double-stranded DNA virus, which include many important human and animal pathogens. Herpes simplex virus 1 (HSV-1) is a contagious neurotropic herpes virus, which encode 12 envelope glycoproteins and play important roles in the viral replication [111–113]. Glycoproteins gB, gC, and gD have been implicated in the attachment of virus to receptors, while gB, gD, and heterodimer gH/gL are essential for HSV attachment and penetration [111,114–119]. HSV-1 gH contains 838 amino acids and has 7 consensus sites for *N*-linked oligosaccharides as well as 11 sites for O-linked glycosylation [120–122]. gL contains 224 amino acids and has one consensus site for *N*-linked oligosaccharides and 3 potential sites for O-linked glycosylation [120,121,123]. HSV-1 gC-1 is heavily glycosylated which contains nine consensus sites for *N*-linked glycosylation and numerous clustered O-linked glycans in a peptide stretch delimited by amino acids 30 and 124 [124]. All the three potential N-glycosylation sites in gD protein are actually glycosylated [125]. Moreover, both gD1 and gD2 are O-glycosylated, and gD2 undergoes greater O-glycosylation compared to gD1 [125]. Two conserved N-linked glycosylation (N48 and N58) sites for gK are critical for virus-induced cell fusion and replication.

Retinoic acid was shown to suppress viral yield in Vero cells and altered *N*-glycosylation of viral envelope proteins [126]. The use of tunicamycin to inhibit *N*-glycosylation of HSV- 1 envelope glycoproteins was associated with the production of noninfectious viral particles [126,127]. Seventy-four O-linked glycosylation sites have been identified on 8 of the 12 HSV-1 envelope proteins [128]. Of the 74 O-glycosylation sites, 34 are localized on the four HSV-1 membrane proteins (gB, gD, gH and gL), which are essential for viral infectivity *in vitro* [128]. Twenty-one glycosylation sites were identified in gB, which is essential for fusion with host cell membranes. The remaining 40 O-glycosites were distributed among four HSV-1 glycoproteins (gC, gE, gI and gG), which are all critical in virus–host interactions and host immune response modulation [128].

Varicella-zoster virus (VZV), a human alphaherpesviruses, encodes various structural glycoproteins that are important for its assembly and trafficking [129,130]. At least seven of its proteins are glycosylated – glycoproteins B, C, E, H, I, K, and L (gB, gC, gE, gH, gI, gK, and gL) [131]. Among them, gE is the most abundant VZV glycoprotein, and its loss leads to impaired viral reproduction [132,133]. Like HSV-1, multiple G proteins have N and O-glycosylation sites, but there are few reports on the specific biological functions of these glycosylation sites.

Other viruses, such as Rabies virus [134], Human Papillomavirus [135], and Metapneumovirus [136] have *N*-linked glycans, which play important roles in infectivity, protein folding, tropism, proteolytic processing, as well as immune escape.

## Glycosylations of nonstructural viral proteins

### Glycosylation and biological functions of flavivirus NS1 protein

Nonstructural viral proteins can also be glycosylated. The flavivirus genome encodes seven NS proteins: NS1, NS2A, NS2B, NS3, NS4A, NS4B, and NS5 [137–139]. DENV NS1 has two glycosylation sites at Asn-130 and Asn-207 [21,140,141] (Figure 1), which are conserved in the family flaviviridae. By destroying the endothelial glycocalyx-like layer (EGL), DENV NS1 has been shown to induce vascular leakage [142]. Mutations of the NS1 protein at the N207 glycosylation site could not lead to EGL degradation or internalization by endothelial cells. Just like DENV, the N207 glycosylation sites of WNV and ZIKV NS1 are essential for internalization. N207 is important in clathrin-mediated endocytosis, a common pathway involved in flavivirus NS1-mediated pathogenesis, where it is important for NS1 endosomal transport. Mutations of Asn-207 slightly delay viral replication [143].

Glycosylation at N130 plays an important role in viral proliferation, NS1 protein secretion, and cytopathic effects. Mutations at the N130 glycosylation site of DENV1 and DENV2 NS1 have been reported to prevent DENV from producing live viruses in mammalian and mosquito cells [144,145]. In DENV4, N130 mutations suppressed viral replication in mammalian and C6/36 cells. The absence of N130 in NS1 inhibited viral neurovirulence in mice models [145–147].

Like DENV, JEV, and ZIKV NS1 have two *N*-linked glycosylation sites at N130 and N207 [148]. Yellow fever (YF) NS1 has glycosylation sites at positions 130 and 208 [149]. In addition, some viruses, such as WNV, St. Louis encephalitis (SLE) and Murray valley encephalitis virus (MVEV) have a third glycosylation site at the 175^th^ amino acid position [150]. Entebbe bat virus (ENTV) has four potential *N*-linked glycosylation sites in NS1, located at Asn-106, Asn-130, Asn-207, and Asn-326 [151] while the tick-borne encephalitis virus (TBEV) NS1 has three putative *N*-linked glycosylation sites at residues 85, 207, and 223 [152]. In brain endothelial cells, WNV and JEV NS1 only interact with the glycocalyx, while YFV NS1 exerts the strongest effects on liver endothelial cells [148]. Multiple amino acid changes in the first glycosylation motif of WNV NS1 protein promoted neuroinvasion in mice models [153]. These findings imply that elucidation of the mechanisms involved in flavivirus NS1 glycosylation is essential for the development of antiviral therapies and NS1-based vaccine approaches.

### Glycosylation and biological functions of coronavirus nonstructural proteins

The nsp3 and nsp4 nonstructural proteins of coronavirus undergo *N*-linked glycosylation in ER. For instance, mouse hepatitis virus MHV nsp3 has a glycosylation site at N1525. The glycosylation site of nsp4 in gamma-coronavirus bronchitis virus (IBV) is at residue N48. For the nsp4 of MHV, two glycosylation sites have been predicted at residues N176 and N237 [76,154]. These two nonstructural proteins play vital roles in the life cycles of coronaviruses; however, the functions of their glycosylation have not been elucidated. Other nonstructural proteins of coronaviruses, such as ORF3a, 8ab, and 3b are also glycosylated, but the significance of these modifications are yet to be determined [155].

In addition, some other viral nonstructural proteins, such as Rotavirus NSP4 [156] and HCV NS4B [157,158], may also be glycosylated, but not discussed in detail here for the limited space of the review.

## Conclusions and perspective

Glycomics is an important study field in cell biology. Viral-related *N*-glycosylation modifications, which confers various advantages on viral survival and virulence, have become a research hotspot. Targeting glycans may be a promising approach for inhibition of viral infections. Not only is glycosylation important for pathogens, it is also vital for hosts. Enveloped viruses hijack host glycosylation mechanisms and take advantage of host-derived glycans to modify their own glycoproteins, which benefits many aspects of viral pathogenesis, including entry into host cells, membrane fusion, and infectivity. Moreover, *N*-linked glycosylation of envelope proteins play important roles in neutralizing antibody sensitivity and immune escape. These glycoproteins are the only antigens expressed on viral surfaces; therefore, they are essential for vaccine development. For some viruses, glycans in nonstructural proteins are vital to their life cycles. Even though the involved mechanisms are yet to be established, their roles should not be underestimated. Elucidating the mechanisms of viral protein glycosylation during viral infection and replication will inform the development of specific antiviral therapies and vaccines.

## Data Availability

Data sharing not applicable to this article as no data sets were generated or analyzed during the current study. Data cited in this review are published and available online or upon request from the authors of the respective publications.
